# ADVANCE CARE PLANNING AMONG LGBT PEOPLE: AN INTEGRATIVE REVIEW AND ANALYSIS

**DOI:** 10.1093/geroni/igz038.647

**Published:** 2019-11-08

**Authors:** Luisa Kcomt, Kevin M Gorey

**Affiliations:** University of Windsor, Windsor, Ontario, Canada

## Abstract

Lesbian, gay, bisexual, and transgender people (LGBT) with advanced illness need culturally competent advanced care planning (ACP) services but often encounter structural and communication barriers. The aim of this study was to examine the ACP behaviors of LGBT people. An integrative rapid review method was used to search electronic databases for peer reviewed and non-peer reviewed publications between 2010 to 2017. Eight survey instruments comprising 30 prevalence estimates were analyzed. ACP discussions between LGBT people and their primary health care providers were rare, with an overall prevalence of 10%. Transgender people were 50–70% less likely than their LGB counterparts to have a living will or to have appointed a healthcare proxy. These results suggest there is a critical need for greater cultural competency among health care providers serving LGBT populations. Social workers can play a key role in advocacy and social justice for LGBT individuals with advanced illness.

